# Identification of genes involved in enhanced membrane vesicle formation in *Pseudomonas aeruginosa* biofilms: surface sensing facilitates vesiculation

**DOI:** 10.3389/fmicb.2023.1252155

**Published:** 2023-12-01

**Authors:** Mizuki Kanno, Takuya Shiota, So Ueno, Minato Takahara, Keisuke Haneda, Yuhei O. Tahara, Masaki Shintani, Ryoma Nakao, Makoto Miyata, Kazuhide Kimbara, Hiroyuki Futamata, Yosuke Tashiro

**Affiliations:** ^1^Graduate School of Science and Technology, Shizuoka University, Hamamatsu, Japan; ^2^Department of Engineering, Graduate School of Integrated Science and Technology, Shizuoka University, Hamamatsu, Japan; ^3^Department of Applied Chemistry and Biochemical Engineering, Faculty of Engineering, Shizuoka University, Hamamatsu, Japan; ^4^Graduate School of Science, Osaka Metropolitan University, Osaka, Japan; ^5^Research Institute of Green Science and Technology, Shizuoka University, Shizuoka, Japan; ^6^Japan Collection of Microorganisms, RIKEN BioResource Research Center, Tsukuba, Japan; ^7^Department of Bacteriology, National Institute of Infectious Diseases, Shinjuku, Tokyo, Japan; ^8^JST PRESTO, Kawaguchi, Japan

**Keywords:** membrane vesicles, biofilm, *Pseudomonas aeruginosa*, Psl, flagellar motility

## Abstract

Membrane vesicles (MVs) are small spherical structures (20–400 nm) produced by most bacteria and have important biological functions including toxin delivery, signal transfer, biofilm formation, and immunomodulation of the host. Although MV formation is enhanced in biofilms of a wide range of bacterial species, the underlying mechanisms are not fully understood. An opportunistic pathogen, *Pseudomonas aeruginosa*, causes chronic infections that can be difficult to treat due to biofilm formation. Since MVs are abundant in biofilms, can transport virulence factors to the host, and have inflammation-inducing functions, the mechanisms of enhanced MV formation in biofilms needs to be elucidated to effectively treat infections. In this study, we evaluated the characteristics of MVs in *P. aeruginosa* PAO1 biofilms, and identified factors that contribute to enhanced MV formation. Vesiculation was significantly enhanced in the static culture; MVs were connected to filamentous substances in the biofilm, and separation between the outer and inner membranes and curvature of the membrane were observed in biofilm cells. By screening a transposon mutant library (8,023 mutants) for alterations in MV formation in biofilms, 66 mutants were identified as low-vesiculation strains (2/3 decrease relative to wild type), whereas no mutant was obtained that produced more MVs (twofold increase). Some transposons were inserted into genes related to biofilm formation, including flagellar motility (*flg*, *fli*, and *mot*) and extracellular polysaccharide synthesis (*psl*). Δ*pelA*Δ*pslA*, which does not synthesize the extracellular polysaccharides Pel and Psl, showed reduced MV production in biofilms but not in planktonic conditions, suggesting that enhanced vesiculation is closely related to the synthesis of biofilm matrices in *P. aeruginosa*. Additionally, we found that blebbing occurred during bacterial attachment. Our findings indicate that biofilm-related factors are closely involved in enhanced MV formation in biofilms and that surface sensing facilitates vesiculation. Furthermore, this work expands the understanding of the infection strategy in *P. aeruginosa* biofilms.

## Introduction

Bacteria possess sophisticated tools to deliver their own substances to other cells. One of the mechanisms is the release of membrane vesicles (MVs), which range in size from 20 to 400 nm and are composed of bacterial outer surfaces such as membrane proteins, phospholipids, and lipopolysaccharides (LPS). MVs facilitate the transfer of nucleic acids and toxic components to other bacteria and host cells, and also play roles in the alteration of biofilm structure, resistance to phages, and release of unnecessary compounds (Tashiro et al., 2012; Toyofuku et al., 2015).

In Gram-negative bacteria, MVs caused by blebbing from the outer membrane (OM) are called outer membrane vesicles (OMVs), and several mechanisms of OMV formation have been identified in different species and culture conditions. Weakened OM-peptidoglycan cross-linking (Suzuki et al., 1978; Deatherage et al., 2009; Schwechheimer et al., 2015), accumulation of misfolded protein in the cell envelope (McBroom and Kuehn, 2007; Tashiro et al., 2009), and accumulation or modification of LPS and phospholipids in the outer leaflet of the OM (Elhenawy et al., 2016; Roier et al., 2016) cause blebbing of the OM. The *Pseudomonas* quinolone signal (PQS; 2-heptyl-3-hydroxy-4-quinolone), which is a quorum-sensing molecule produced by *Pseudomonas aeruginosa* (Pesci et al., 1999), is also known to stimulate OMV production in not only *P. aeruginosa* but also other bacteria (Mashburn and Whiteley, 2005; Tashiro et al., 2010a) by insertion into the outer leaflet and OM curvature (Schertzer and Whiteley, 2012). Explosive cell lysis (ECL) is another pathway of MV formation that is distinct from blebbing (Turnbull et al., 2016). ECL is triggered by endolysin, which degrades peptidoglycan, causing cell death and the release of large amounts of MVs.

The relationship between MVs and biofilms has been the focus of research for decades, as MVs are a component of biofilms formed under natural and experimental conditions (Beveridge et al., 1997; Schooling and Beveridge, 2006; Grande et al., 2015; Wang et al., 2015; Baeza and Mercade, 2020). However, research on MV biogenesis has been mainly conducted in a planktonic state. According to a report by the National Institutes of Health (NIH), among all microbial and chronic infections, 65 and 80%, respectively, are associated with biofilm formation (Jamal et al., 2018). Pathogenic bacteria cause infections by forming chronic biofilms that persist in mucus-rich environments (Costerton et al., 1999). Biofilms form in the lungs, cornea, and skin of individual animals, contributing to the initiation and spread of infection, as well as in the soil, hospital surfaces, and many other locations in the environment (Hall-Stoodley et al., 2004). Therefore, a comprehensive understanding of the function and formation mechanisms of MVs in biofilms is important for both clinical and environmental conditions.

The biofilm matrix, which helps protect biofilm-embedded cells from external environmental stress and threats (Hobley et al., 2015), is mainly composed of exopolysaccharides, extracellular proteins, eDNA, and MVs. Several studies have suggested that MVs promote biofilm formation in *Helicobacter pylori* (Yonezawa et al., 2009), *Pseudomonas putida* (Baumgarten et al., 2012), *Vibrio cholerae* (Altindis et al., 2014), and *P. aeruginosa* (Murphy et al., 2014), and also function to facilitate dispersion in *Xylella fastidiosa* (Ionescu et al., 2014) and *P. aeruginosa* (Esoda and Kuehn, 2019; Cooke et al., 2020). The MVs derived from planktonic and biofilm states are quite different in physical characteristics and proteomics in several bacteria, including *P. aeruginosa* (Schooling and Beveridge, 2006; Schooling et al., 2009; Toyofuku et al., 2012; Park et al., 2014b,^2015^; Couto et al., 2015), *H. pylori* (Grande et al., 2015), *Lactobacillus reuteri* (Grande et al., 2017), and *Bordetella pertussis* (Carriquiriborde et al., 2021). Based on these studies, the biogenesis and function of MVs in the biofilm state are considered to be different from those in the planktonic state.

*Pseudomonas aeruginosa* is a prominent opportunistic human pathogen that establishes chronic biofilm-based infections, such as cystic fibrosis, and is used as a model organism for biofilm formation. MV production by several bacteria, including *P. aeruginosa*, is increased in biofilms (Schooling and Beveridge, 2006; Grande et al., 2017), but the mechanisms have not been fully elucidated. *P. aeruginosa* induces host immune responses by releasing large amounts of MVs that retain antigens in the human body (Bauman and Kuehn, 2009; Bomberger et al., 2009; Ellis et al., 2010). Therefore, understanding the MV production mechanism under biofilm conditions leads to control of biofilm formation and bacterial infections.

Although biofilm growth mode is predominant in natural and disease environments, most studies on the function and mechanism of MV formation have been conducted using planktonic cultures. In this study, we aimed to unravel the molecular mechanism behind enhanced MV formation under biofilm conditions in *P. aeruginosa*. Liquid static biofilm was used for the biofilm model because MV formation was significantly promoted compared to planktonic conditions and is a suitable model for studying the relationship between attachment and MV formation. Transposon mutagenesis screens were performed to identify genes that affect enhanced MV formation in *P. aeruginosa* PAO1 biofilm. Many mutants lacking biofilm-related genes were identified as low-vesiculating mutants; some mutants (e.g., those related to flagellar motility and Psl synthesis) showed low-biofilm-forming abilities, whereas others showed low vesiculation even though biofilm formation was not repressed. We also showed that the initial attachment triggers vesiculation. These results provide novel insights into how bacteria produce MVs in clinically relevant biofilms.

## Materials and methods

### Microbial strains, plasmids, and growth conditions

The bacterial strains, plasmids, and primers used in this study are listed in Table 1 and Supplementary Table 1. *P. aeruginosa* and *Escherichia coli* were routinely grown at 37°C aerobically in Miller’s lysogeny broth (LB) (1% w/v tryptone, 0.5% w/v yeast extract, and 1% w/v NaCl). *P. aeruginosa* PAO1 and its mutants were inoculated to adjust an optical density at 600 nm of 0.02, and grown at 37°C for 12 h in either a shaking condition (planktonic condition: 100 ml LB medium in each flask at 200 rpm) or a static biofilm condition (10 ml LB medium in each petri dish without shaking). When required, the following antibiotics were used: 20 μg/ml tetracycline or 10 μg/ml gentamicin for *E. coli*, and 60 μg/ml tetracycline or 100 μg/ml gentamicin for *P. aeruginosa*. 2,6-Diaminopimelic acid (DAPA) was added to the medium at a concentration of 300 μM for the growth of *E. coli* β2163.

**TABLE 1:** Strains, plasmids used in this study.

Strains and plasmids	Genotype or description	References
***P. aeruginosa***		
PAO1	Wild type	Holloway et al., 1979
Δ*pelA*	PAO1 Δ*pelA* mutant	Tashiro et al., 2014
Δ*pslA*	PAO1 Δ*pslA* mutant	Tashiro et al., 2014
Δ*pelA*Δ*pslA*	PAO1 Δ*pelA*Δ*pslA* mutant	Tashiro et al., 2014
*flgK*::T8	Tn T8 was inserted	This study
*motA*::T8	Tn T8 was inserted	This study
***E. coli***		
β2163	(F^–^) PR4-2-Tc::Mu Δ*dapA*::(*erm*-*pir*) [Km*^r^* Em^*r*^]	Demarre et al., 2005
**Plasmids**		
pIT2	Plasmid containing ISlacZ/hah transposon derived from Tn5	Jacobs et al., 2003
pBBR1MCS-5	Broad host range vector, Gm^r^, *lacZ*α	Kovach et al., 1995
pPslA	Comprising the gene *pslA* in the HindIII-XbaI region of pBBR1MCS-5	This study

### Extraction, purification, and quantification of vesicles

Vesicle extraction from *P. aeruginosa* culture was performed by ultracentrifugation of the filtered supernatant, as previously described (Takaki et al., 2020). *P. aeruginosa* was grown under shaking and static conditions overnight. In static biofilm conditions, the mucoid and viscous films attached to the bottom of petri dish were scratched using a scraper. The collected cultures were treated by sonication (38 kHz self-oscillation for 5 min) and vortex to isolate vesicles from the bacterial cell surfaces. After the removal of bacterial cells using centrifugation (6,000 × *g* for 15 min at 4°C), the supernatant was sequentially filtered through 0.45 and 0.20 μm pore-size membranes (CELLULOSE ACETATE, ADVANTEC) and ultracentrifuged at 150,000 × *g* for 2 h at 4°C using a Himac CP80WX and a P45AT angle rotor (Eppendorf Himac Technologies). The supernatant was removed, and the pellets were resuspended in 50 mM HEPES (pH 6.8) and 0.85% NaCl (HEPES-NaCl buffer).

Purification of extracted vesicles was conducted by density gradient ultracentrifugation using different layered concentrations of iodixanol, as previously described (Takaki et al., 2020). Briefly, the extracted vesicles were adjusted to 1 ml of 45% (wt/vol) iodixanol (OptiPrep; Axis-Shield Diagnostics Ltd.) in HEPES-NaCl, transferred to the bottom of ultracentrifuge tubes, and layered with iodixanol-HEPES-NaCl (2 ml of 40, 35, 30, and 25% and 1 ml of 20%). The samples were ultracentrifuged at 100,000 × *g* for 16 h at 4°C using a swinging bucket rotor (P40ST; Eppendorf Himac Technologies). The MV fraction was further ultracentrifuged at 200,000 × *g* for 1 h at 4°C using a P50A3 rotor, and the pellet was resuspended in HEPES-NaCl to remove excess iodixanol.

Quantification of vesicles using FM4-64 was followed by a previous report (Aktar et al., 2021). Briefly, to extract vesicles, the cell-free supernatant was ultracentrifuged at 200,000 × *g* for 1 h at 4°C using a P50A3 rotor. The extracted vesicles were incubated with 10 μg/ml of FM4-64 (Biotium, Inc.) for 30 min on a 96-well black plate at 37°C in the dark, and the fluorescence intensities were measured using a microplate reader (SpectraMax i3, Molecular Devices) at 506/750 nm (excitation/emission wavelength). Linoleic acid was used as a standard, and the value was presented as the phospholipid amount in vesicles normalized to the cell density.

### Phase contrast, fluorescence microscopic observations

Phase contrast, fluorescence microscopic observations were conducted using an Olympus IX73 microscope (Olympus), and images were captured with a scientific complementary metal oxide semiconductor (sCMOS) camera (Andor). The excitation light was emitted using a mercury lamp (U-HGLGPS, Olympus). The cell membrane images were recorded using the GWA filter set [Ex/Em (nm), 530–550/575–625], and the polysaccharide Psl images were recorded using the BNA filter set [Ex/Em (nm), 470–495/510–550]. The phase contrast and fluorescence channels were adjusted using the microscope imaging software CellSens Dimension (Olympus).

For phase contrast and fluorescence observation of bacterial cells attached to the glass, 18 × 18 mm coverslips were placed in a 6-well plate with 3 ml of the *P. aeruginosa* culture and OD_600_ adjusted to 0.1. Only half of the coverslips were then immersed. The cells were statically grown at 37°C for 2.5 h. Unattached cells were removed from coverslips after incubation by rinse with 1 ml of 1 × PBS. Psl was detected by labeling with 2 μg of HHA-FITC lectin (Hippeastrum Hybrid, EY Laboratories, Inc.) for 20 min at 25°C in the dark. Bacterial cells on coverslips were then stained with 10 μg/ml FM4-64 (SynaptoRed C2, Biotium, Inc.) for 10 min at 37°C in the dark. To observe free-living cells, planktonic bacterial cells in a 6-well plate were collected and centrifuged (6,000 × *g* for 2 min at room temperature), then cells were stained with FM4-64 as described.

### Electron microscope observation

For scanning electron microscope (SEM) observation, vesicles or bacterial cells were prepared on attached glasses. For vesicles and planktonic bacterial cells, samples were dropped onto glasses coated with 0.5% poly-L-lysine hydrobromide (Sigma-Aldrich). In the case of biofilms, two small coverslips were prepared: one was vertically stood and fixed with tape, and the other was laid at the bottom in the static culture. Fixation, dehydration through ascending acetone series, critical point drying, and osmium coating were conducted using previously reported methods (Tashiro et al., 2008). The samples were observed using SEM Regulus 8220 (Hitachi High-Tech).

Transmission electron microscope (TEM) observations of negatively stained vesicles and quick-freeze deep-etched (QFDE) bacterial cells were performed as previously described (Takaki et al., 2020) using a JEM-1010 (JEOL) equipped with a FastScan-F214 (T) CCD camera (TVIPS) or JEM-2100F at 80 kV with a CANTEGA G2 CCD camera (Olympus), or CANTEGA G2 (Olympus), respectively. To prepare samples for negative staining, Cu400 mesh grids (JEOL) were pretreated with 0.01% α-poly-L-lysine, vesicles were placed onto the grid, and samples were stained with 2% (NH_4_)_6_Mo_7_O_24_. For QFDE sample preparation, bacterial cells grown under shaking or static conditions were centrifuged; after washing, they were mixed with a rabbit lung slab and mica flakes. The mixture was placed on a paper disk attached to an aluminum disc. The samples were quickly frozen in liquid helium using a CryoPress (Valiant Instruments) and maintained at −180°C using a JFDV freeze-etching device (JEOL). The samples were freeze-fractured with a knife, freeze-dried, and coated with platinum and carbon. After floating in full-strength hydrofluoric acid and rinsing in water, the replica was cleaned with a commercial bleach containing sodium hypochlorite. The replica specimens were rinsed and placed on the grids.

### Nanoparticle tracking analysis

The size of vesicles was measured using ZetaView PMX 120 (Particle Metrix). Polystyrene nanospheres (100 nm) were used to assess the measurement bias of size and concentration. Vesicles were diluted to allow for 50–200 particles in 1 frame.

### Protein composition analysis

Membrane vesicles were purified using the density gradient centrifugation method described above, except that a protein inhibitor was added to all solvents at appropriate concentrations in each step. Bacterial OMPs were isolated from lysed total cell proteins by Sarkosyl separation, as described previously (Tashiro et al., 2008). SDS-PAGE and peptide mass fingerprinting (PMF) were performed as reported previously (Takaki et al., 2020).

### Transposon mutagenesis and identification of transposon insertion location

Transposon insertions in the PAO1 chromosome were generated as previously reported (Jacobs et al., 2003), with some modifications. Transposon insertions in the PAO1 chromosome were generated by mating *P. aeruginosa* PAO1 with β2163/pIT2 (IS*lacZ*/*hah* insertions).

Mating was performed for 4 h at 37°C on LB agar containing DAPA. Mutagenized cells were selected on LB agar containing 60 μg/ml tetracycline, 20 μg/ml chloramphenicol, and 40 μg/ml 5-bromo-4-chloro-3-indolyl-β-D-galactoside (X-gal; Wako) and used for screening.

The screening of strains with altered vesicle production was performed through two steps. In the first screening, *P. aeruginosa* wild-type strain and transposon mutants were inoculated in 200 μl of LB culture in 96-well plates and grown statically at 37°C for 16 h. To obtain colonies, cell cultures were plated on square LB agar plates containing 60 μg/ml tetracycline using a 96-pin microplate replicator and incubated overnight at 37°C. The colonies were temporarily stored at 4°C. After measuring OD_600_, cultures were transferred to 96-well PCR plates (FastGene Ultra Easy Cut; NIPPON Genetics) and centrifuged at 2,576 × *g* for 30 min at 4°C to remove cells. The supernatant (50 μl) was mixed with 50 μl of 10 μg/ml FM4-64 in fresh 96-well plates and incubated at 37°C for 30 min in the dark. Fluorescence was measured at 506/750 nm (excitation/emission) using a microplate reader. Fresh LB was used as the standard. Vesicle production was expressed as the amount of phospholipid normalized to the cell density. In the second screening, transposon mutants that showed alterations in MV production were used, and vesicle production was measured in triplicate as in the first screening.

Transposon insertion sites were determined according to a previous study (Jacobs et al., 2003) with some modifications. Two-stage PCR and sequencing were performed. For PCR experiments, KOD One PCR Master Mix (TOYOBO) and primer pairs (lacZ-211, CEKG2A, CEKG2B, CEKG2C for the first round, and lacZ-148 and CEKG4 for the second round) were used to amplify the DNA fragments. In the first round of PCR, the colony suspension was used as a template. In the second round, the PCR products from the first round were used as templates. The PCR products from the second round were cleaned up by using the FastGene Gel/PCR Extraction Kit (NIPPON Genetics Co., Ltd.) and used as sequencing templates with the primers shown in Supplementary Table 1. Sequencing analysis was performed by Eurofins.

### Plasmid construction

A DNA fragment containing the open reading frame of *pslA* was amplified by PCR using primers pslA-F-Hind3 and pslA-R-Xba1. A broad-host-range vector pBBR1MCS-5 was digested with HinIII and XbaI, and the *pslA* DNA fragment was inserted into pBBR1MCS-5 by Gibson Assembly (New England Biolabs), yielding plasmid pPslA. Plasmids were transformed into PAO1 and Δ*pslA*.

### Crystal violet biofilm assay

To assess cell attachment, 96-well microtiter plates were inoculated with 200 μl of culture in LB at an OD_600_ of 0.02 and statically incubated at 37°C for 6 h. Biomass was quantified by crystal violet (CV) staining according as reported previously (O’Toole and Kolter, 1998). After measuring the OD_600_ using a microplate reader (SpectraMax i3, Molecular Devices), the supernatant was removed, and the wells were washed twice with 250 μl of distilled water. The plates were allowed to dry and 225 μl of 0.1% CV was added to each well. The plates were then incubated at room temperature. Following staining, the wells were washed thrice with 300 μl of distilled water to remove excess CV. After drying, 250 μl of a 4:1 ethanol:acetone solution was added to each well to solubilize the CV, and the absorbance was measured at 570 nm. The absorbance per OD_600_ was defined as the biofilm formation per cell.

### Calculation of specific growth rate

PAO1 wild-type and transposon mutants were inoculated to 200 μl of LB at an initial OD_600_ of 0.02 in 96-well microtiter plates and grown at 37°C at 200 rpm. Growth curves were measured every hour, and their specific growth rates μ [h^–1^] were calculated as follows:


$$\mu =\text{l}\,n\,\frac{{\text{X}}_{i}}{{\text{X}}_{0}}/\left({\text{t}}_{i}-{\text{t}}_{0}\right)$$

*X*_i_ and *X*_0_ are cell densities measured at 600 nm at each time. *t*_*i*_ and *t*_0_ indicate the respective time [h].

### Statistical analysis

For all experiments, results were calculated from more than three biological replicates and the average ± standard deviation (SD) is shown. Statistical analyses were conducted using Student’s *t*-tests or one-way ANOVA for two groups or three groups, respectively.

## Results

### Vesicle production and properties in liquid static culture

**FIGURE 1:**
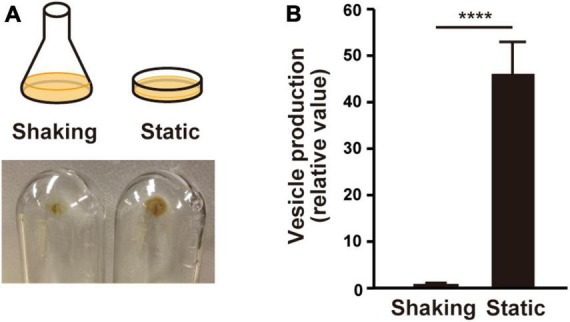
Vesicle formation is increased in liquid static culture in *P. aeruginosa* PAO1. **(A)** Graphical representation of culture conditions (upper) and visualization of ultracentrifuged pellets from the supernatant of the cultures from shaking (planktonic) and liquid static (biofilm) conditions after 12 h (lower). **(B)** Vesicle formation of PAO1. The amount of vesicles extracted from the supernatants was normalized to the cell density, and each value shown is relative to that of the shaking condition. The data are presented as the mean ± SD from three replicates. ****P < 0.0001 (*t*-tests).

It is commonly recognized that MV formation increases under biofilm conditions in many bacteria. In this study, we used static culture, in which PAO1 was grown in LB liquid medium in a petri dish as a biofilm model, in contrast to a flask shaking culture as planktonic condition. After incubation overnight, the static culture medium was mucoid, and a viscous film was attached to the bottom. Whole cells and viscous films were scratched from the petri dish, and MVs contained in the supernatant were isolated by ultracentrifugation after cell removal. The ultracentrifuge pellet from the static culture was greater than that from the shaking culture (Figure 1A). Quantification of vesicles with lipid probe FM4-64 revealed that vesicle production was significantly enhanced in the static culture compared to that in the shaking culture (Figure 1B). These results indicate that vesiculation is enhanced in the static culture.

**FIGURE 2:**
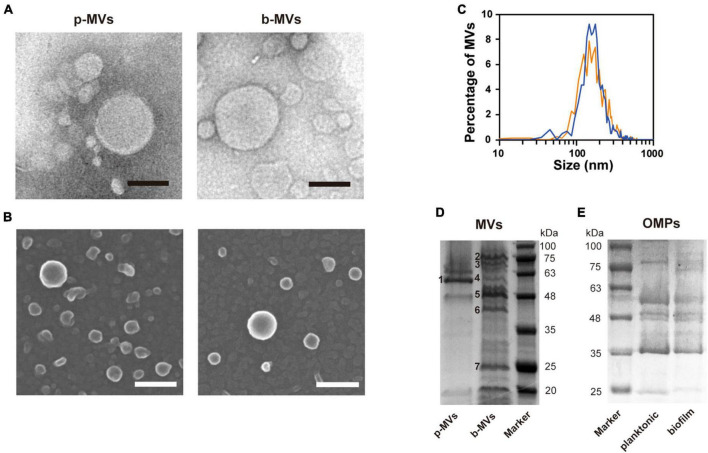
Characteristics and protein patterns of p-MVs and b-MVs. **(A)** TEM observation of negatively stained purified MVs. Bar, 100 nm. **(B)** SEM observation of purified MVs. Bar, 100 nm. **(C)** Nano-tracking analysis of p-MVs (orange) and b-MVs (blue). Protein patterns of MVs **(D)** and OMPs **(E)** separated by SDS-PAGE with Coomassie brilliant blue. Inset numbers indicate the identified bands shown in Table 2.

We named the MVs formed under shaking and static culture conditions planktonic MVs (p-MVs) and biofilm MVs (b-MVs), respectively. p-MVs and b-MVs were purified using gradient ultracentrifugation from crude MVs and visualized by TEM and SEM. The results showed no significant changes in their appearance (Figures 2A, B). Nanoparticle tracking analysis (NTA) also showed that the average diameters and distributions between p-MVs and b-MVs were not significantly different (Figure 2C). These results indicate no significant differences in size and shape between p-MVs and b-MVs.

**TABLE 2:** Major proteins identified in MVs.

Band no.^a^	Size (kDa)	GenBank	Mascot score	PA number	Protein name
1	57.8	AAG06327.1	167	PA2939	Probable aminopeptidase PaAP
2	80.0	AAG07609.1	117	PA4221	Fe (III)-pyochelin receptor FptA
3	63.1	AAG07310.1	120	PA3923	Unknown OMP
4	50.4	AAG06923.1	138	PA3535	Probable serine protease precursor EprS
5	46.9	AAG04347.1	111	PA0958	OM porin OprD
6	44.9	AAG06426.1	100	PA3038	OM porin OpdQ
7	22.0	AAG08209.1	110	PA4824	Unknown

^a^Numbers corresponds to inset them in Figure 2D.

To further investigate whether different growth conditions alter the components of MVs, purified p-MVs and b-MVs with protease inhibitors were separated by SDS-PAGE, and the protein profiles were compared. Some major bands were excised and identified using PMF. Although a major component of p-MVs was leucine aminopeptidase PaAP, consistent with previous reports (Bauman and Kuehn, 2006; Tashiro et al., 2010b), the protein pattern of b-MVs was significantly different (Figure 2D). FptA, PA3923, Esp, OprD, OpdQ, and PA4824 were the major protein components of b-MVs (Table 2). However, no significant differences in the OMPs between planktonic and biofilm cells were observed (Figure 2E), suggesting that the different distribution of proteins in b-MVs is not due to altered OMP profiles. These results indicate that a specific mechanism may induce MV production in the static biofilm condition.

### Vesicles are associated with filamentous substances in static biofilm

**FIGURE 3:**
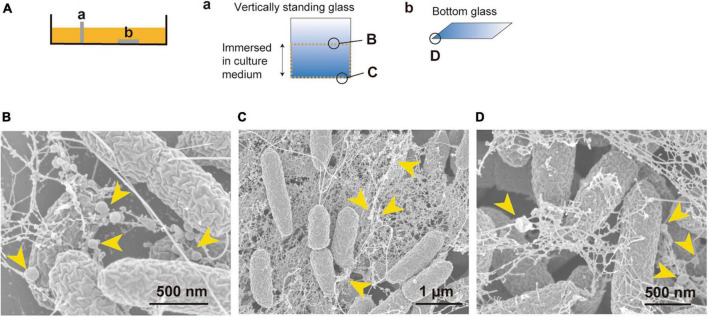
Vesicles are a biofilm component and are associated with filamentous structures in PAO1 liquid static culture. **(A)** Cover glasses were either vertically standing (a) or placed at the bottom of the static culture (b). Observation areas of B, C, and D are shown in circles. **(B–D)** Bacterial cells inside biofilm visualized by SEM. The air-liquid interface **(B)** and the bottom **(C)** of the vertically standing glass, and the edge of the glass placed at the bottom of the static culture **(D)** are shown. Vesicles are attached to filamentous structures and are present as a component of the biofilm (yellow arrows). Additional images are shown in Supplementary Figure 1.

It was of interest to investigate what is the state of the bacteria and how b-MVs interact with bacterial cells in the static culture. We prepared two glasses in the liquid static culture; one was placed vertically, and the other was placed horizontally at the bottom of the culture, and after incubation overnight were visualized using SEM (Figure 3A). On the glass placed vertically, thick biofilms were observed at the air-liquid interface (Figure 3B and Supplementary Figures 1A–D) and the bottom edge of the glass (Figure 3C and Supplementary Figures 1E–G). Fibrous structures formed connections between cells, and b-MVs were observed on both the glass and fibrous structures (Figures 3B, C). These fibrous structures are considered as Psl exopolysaccharides and flagella in PAO1 biofilms according to a previous report (Byrd et al., 2010). A thick biofilm was also observed on the edge of the glass placed horizontally (Figure 3D and Supplementary Figures 1H, I), and b-MVs were intertwined with fibrous structures (Figure 3D). On the other hand, that fibrous structures were not observed on the cell surfaces grown in shaking culture (Supplementary Figure 1J). These results indicate that high MV production is related to biofilm formation in static culture and that MVs function as biofilm components.

### Curvature of the outer membrane in static biofilm conditions

**FIGURE 4:**
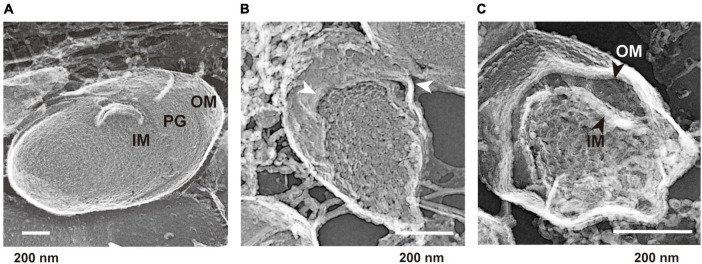
Quick-freeze deep-etch and replica electron micrographs of *P. aeruginosa* PAO1 cells. **(A)** An image of cells grown under the shaking condition for 12 h. Outer membrane (OM), inner membrane (IM), and peptidoglycan (PG) are shown at the cross sections. **(B,C)** Images of cells grown under the static condition for 12 h. The protrusion of the OM is shown by white arrows **(B)**. A broad space between the OM and IM (black arrows) was observed in some cells **(C)**. Additional images are shown in Supplementary Figure 2.

We hypothesized that the surface structures in static culture are different from those in the shaking condition, and that these alterations induce b-MV formation. To investigate the spatial structure of the bacterial envelope, we applied quick-freeze deep-etch and replica electron microscopy (QFDE-EM) method to visualize the cellular surface with high spatial and sub-millisecond time resolutions. When cells were grown under shaking conditions, the intracellular compartments, including the presumptive OM, peptidoglycan (PG) and inner membrane (IM) were clearly visualized in the freeze-fractured sections (Figure 4A and Supplementary Figures 2B, C). Cell sections from the static condition showed different surface characteristics; some curvatures of the OM were observed at the surface (Figure 4B and Supplementary Figures 2E, F), suggesting that there are vesicles released from the protrusion of the OM in static biofilm conditions. Furthermore, the OM and IM were separated, and large periplasmic spaces were observed in some cells (Figure 4C and Supplementary Figure 2F); these vesicles are therefore considered to be composed of the OM as a result of blebbing. b-MVs observed under this condition seemed to be released due to alteration of cell envelope structure.

### Identification of genes related to MV formation in biofilms

To identify the factors involved in increased MV formation in biofilms, we constructed a *P. aeruginosa* mutant library using transposon T8 derived from Tn5. The 8,023 transformants obtained were statically grown in LB medium with microtiter plates, and MV production was analyzed with the lipid probe FM4-64. After the first and second screenings, 66 mutants were identified as low-vesiculation strains (2/3 decrease relative to wild type), whereas no mutant was obtained that produced more MVs (twofold increase). The transposon insertion sites of low MV-producing mutants were determined by sequencing and are shown in Table 3.

**TABLE 3:** Summary of MV production and mutated genes in transposon mutants.

Class and mutant number	Locus tag (sequence length after Tn insertion^a,b^)	Mutated gene	Function	Relative MV production^c^	Relative biofilm formation^d,e^	Specific growth rate [h^–1^]^f^
PAO1 wild type	–	–	–	1.0 (±0.0084)	1.0 (±0.49)	1.6
Flagella synthesis and motility						
PAMK87B4	PA1082 (1169022)	*flgG*	Flagellar basal-body rod protein	0.31 (±0.047)	0.019 (±0.010)	0.91
PAMK81E8	PA1083 (1169338)	*flgH*	Flagellar L-ring protein precursor	0.092 (±0.044)	0.011 (±0.0044)	0.88
PAMK8A8	PA1084 (1170369)	*flgI*	Flagellar P-ring protein precursor	0.14 (±0.18)	0.59 (±0.27)	1.0
PAMK91D4	PA1085 (1171009)	*flgJ*	Flagellar protein	0.21 (±0.11)	b.d.	0.86
PAMK28D5	PA1086 (1173097)	*flgK*	Flagellar hook-associated protein 1	0.28 (±0.089)	0.014 (±0.010)	0.91
PAMK58F3	PA1086 (1173441)	*flgK*	Flagellar hook-associated protein 1	0.17 (±0.043)	0.0088 (±0.011)	1.0
PAMK31A8	PA1087 (1175341)	*flgL*	Flagellar hook-associated protein type 3	0.24 (±0.13)	b.d.	0.93
PAMK54D7	PA1087 (1175503)	*flgL*	Flagellar hook-associated protein type 3	0.26 (±0.047)	b.d.	0.88
PAMK78C3	PA1094 (1185597)	*fliD*	Flagellar capping protein	0.17 (±0.075)	0.014 (±0.0065)	0.84
PAMK96F7	PA1094 (1185257)	*fliD*	Flagellar capping protein	0.21 (±0.012)	b.d.	0.92
PAMK55E2	PA1104 (1196515)	*fliI*	Flagellum-specific ATP synthase	0.24 (±0.048)	0.030 (±0.012)	0.92
PAMK9C8	PA1441 (1571030)	*fliK*	Flagellar hook-length control protein	0.080 (±0.048)	0.65 (±0.23)	1.1
PAMK6A10	PA1448 (1576229)	*fliR*	Role in flagellar biosynthesis	0.041 (±0.040)	0.044 (±0.039)	0.92
PAMK78H4	PA1454 (1584113)	*fleN*	Flagellar synthesis regulator	0.11 (±0.024)	0.090 (±0.067)	1.1
PAMK98G9	PA1455 (1584890)	*fliA*	Sigma factor	0.072 (±0.016)	0.015 (±0.0081)	0.90
PAMK20F10	PA1460 (1590807)	*motC*	Flagellar motor protein	0.24 (±0.024)	0.47 (±0.19)	1.0
PAMK80C12	PA1461 (1591726)	*motD*	Flagellar motor protein	0.088 (±0.030)	0.021 (±0.011)	0.94
PAMK94C10	PA1461 (1591361)	*motD*	Flagellar motor protein	0.17 (±0.082)	0.019 (±0.010)	1.0
PAMK57A8	PA3526 (3946680)	*motY*	Sodium-type flagellar protein	0.13 (±0.061)	0.029 (±0.0086)	1.3
PAMK91B7	PA3526 (3946715)	*motY*	Sodium-type flagellar protein	0.20 (±0.080)	0.024 (±0.0094)	1.2
PAMK37G5	PA4954 (5559399)	*motA*	Chemotaxis protein	0.28 (±0.22)	0.025 (±0.013)	1.0
Metabolic pathway						
PAMK81F2	PA0401 (443506)		Noncatalytic dihydroorotase-like protein	0.17 (±0.0076)	2.1 (±0.58)	1.0
PAMK74A4	PA0945 (1033412)	*purM*	Phosphoribosylaminoimidazole synthetase	0.10 (±0.0099)	0.97 (±0.30)	1.1
PAMK77E2	PA1013 (1096471)	*purC*	Phosphoribosylaminoimidazole-succinocarboxamide synthase	0.11 (±0.011)	1.1 (±0.44)	1.0
PAMK75E5	PA1375 (1492714)	*pdxB*	Erythronate-4-phosphate dehydrogenase	0.11 (±0.021)	2.5 (±0.96)	1.0
PAMK73D10	PA2013 (2203019)	*liuC*	Putative 3-methylglutaconyl-CoA hydratase	0.31 (±0.047)	0.67 (±0.31)	1.3
PAMK11H7	PA2014 (2204985)	*liuB*	Methylcrotonyl-CoA carboxylase, beta-subunit	0.20 (±0.0049)	0.71 (±0.29)	1.3
PAMK54D11	PA2014 (2204172)	*liuB*	Methylcrotonyl-CoA carboxylase, beta-subunit	0.25 (±0.023)	1.6 (±0.58)	1.5
PAMK5B11	PA3584 (4017261)	*glpD*	G3P dehydrogenase	0.088 (±0.025)	0.92 (±0.39)	1.5
PAMK39D2	PA3763 (4218398)	*purL*	Phosphoribosylformylglycinamidine synthase	0.090 (±0.016)	0.82 (±0.47)	1.4
PAMK34G7	PA3916 (4386135)	*moaE*	Molybdopterin converting factor, large subunit	0.20 (±0.098)	0.80 (±0.36)	1.4
PAMK94F9	PA4640 (5208591)	*mqoB*	Malate:quinone oxidoreductase	0.33 (±0.068)	1.5 (±0.55)	1.1
PAMK33F8	PA4855 (5451843)	*purD*	Phosphoribosylamine–glycine ligase	0.33 (±0.12)	0.87 (±0.32)	1.6
PAMK78D2	PA5426 (6106706)	*purE*	Phosphoribosylaminoimidazole carboxylase, catalytic subunit	0.12 (±0.019)	1.2 (±0.38)	1.0
Exopolysaccharide synthesis						
PAMK82C6	PA2235 (2458813)	*pslE*	Transporters	0.16 (±0.049)	0.068 (±0.061)	1.3
PAMK81A10	PA2239 (2464156)	*pslI*	Glycosyltransferases	0.12 (±0.0071)	b.d.	1.2
PAMK82F5	PA2241 (2467086)	*pslK*	Glycosyltransferases	0.25 (±0.075)	0.14 (±0.060)	1.4
PAMK95D8	PA2242 (2468936)	*pslL*	Acetylase	0.32 (±0.13)	b.d.	1.2
PAMK102E9	PA5322 (5992772)	*algC*	Phosphomannomutase	0.15 (±0.065)	2.1 (±0.37)	1.0
Two component system						
PAMK36F3	PA0413 (460798)	*chpA*	Component of chemotactic signal transduction system	0.30 (±0.067)	0.78 (±0.28)	1.1
PAMK14C4	PA1099 (1190543)	*fleR*	Two component response regulator protein	0.30 (±0.096)	0.048 (±0.041)	0.91
PAMK43B8	PA1099 (1191376)	*fleR*	Two component response regulator protein	0.23 (±0.086)	b.d.	0.89
PAMK49C9	PA1611 (1755445)		Two component system histidine kinase	0.18 (±0.044)	0.70 (±0.25)	1.3
PAMK18A3	PA3346 (3758366)	*hsbR*	Two-component response regulator	0.29 (±0.037)	0.71 (±0.23)	1.3
PAMK83D4	PA4493 (5028482)	*roxR*	DNA binding response regulator, two component response regulator	0.26 (±0.041)	0.68 (±0.24)	1.4
PAMK43H10	PA5484 (6175941)	*kinB*	Histidine kinase	0.21 (±0.050)	0.99 (±0.45)	1.5
Signal synthesis						
PAMK101F5	PA0169 (193200)	*siaD*	The diguanylate cyclase	0.054 (±0.032)	0.063 (±0.019)	1.8
PAMK25E10	PA0170 (193932)	*siaC*	Putative anti-sigma factor antagonist	0.26 (±0.066)	0.054 (±0.024)	1.5
PAMK78G2	PA0744 (812073)		Probable enoyl-CoA hydratase/isomerase	0.078 (±0.0094)	0.85 (±0.12)	1.3
PAMK90H12	PA0745 (813068)	*dspI*	Putative enoyl-CoA hydratase/isomerase	0.23 (±0.16)	0.78 (±0.12)	1.7
PAMK20E7	PA1430 (1558495)	*lasR*	Transcriptional regulator	0.24 (±0.017)	1.3 (±0.33)	1.7
PAMK42F4	PA3479 (3892951)	*rhlA*	Rhamnosyltransferase chain A	0.13 (±0.028)	0.70 (±0.21)	1.6
PAMK92G8	PA3479 (3892627)	*rhlA*	Rhamnosyltransferase chain A	0.20 (±0.031)	0.60 (±0.13)	1.5
Transcriptional						
PAMK86E7	PA1097 (1188773)	*fleQ*	Transcriptional regulator	0.29 (±0.061)	b.d.	1.4
PAMK33F4	PA2736.1 (3099361)		tRNA-Pro	0.33 (±0.044)	0.79 (±0.13)	1.5
PAMK76C11	PA4745 (5330112)	*nusA*	N utilization substance protein A	0.26 (±0.078)	0.10 (±0.020)	1.4
PAMK89B9	PA4755 (5339489)	*greA*	Transcription elongation factor	0.084 (±0.031)	0.56 (±0.11)	1.8
PAMK81D12	PA4756 (5341379)	*carB*	Carbamoyl phosphate synthetase large subunit	0.055 (±0.013)	1.3 (±0.17)	1.0
Unclassified						
PAMK81G5	PA1714 (1858225)	*exsD*	T3SS antiactivator protein	0.30 (±0.11)	1.2 (±0.32)	1.8
PAMK15F2	PA2228 (2449582)		Beta-lactamase domain-containing protein	0.26 (±0.050)	0.91 (±0.18)	1.6
PAMK89A9	PA2228 (2450402)		Beta-lactamase domain-containing protein	0.25 (±0.038)	0.90 (±0.29)	2.1
PAMK95B11	PA3978 (4459108)			0.29 (±0.062)	1.0 (±0.17)	2.0
PAMK69H2	PA4005 (4486111)	*rsfS*	Ribosomal silencing factor	0.23 (±0.070)	1.1 (±0.085)	1.5
PAMK98D4	PA5565 (6258000)	*gidA*	Glucose-inhibited division protein A	0.28 (±0.042)	3.4 (±0.78)	1.0
PAMK38E2	(4965081)			0.14 (±0.052)	1.4 (±0.24)	1.6
PAMK51A7	(6198048)			0.23 (±0.0058)	1.1 (±0.23)	1.9

^a^Tn, transposon.

^b^Transposon insertion sites were identified by alignment with the nucleotide sequence of P. aeruginosa PAO1 GenBank reference (RefSeq accession number, NC_002516). The length of the PAO1 whole genome sequence is 6,264,403 bp.

^c^MV production values are from the results of the second screening. The data are presented as the mean ± SD from three replicates.

^d^The data are presented as the mean ± SD from five replicates.

^e^b.d., below detection.

^f^The growth curves were obtained from triplicates and each specific growth rate (μ) was calculated.

Many of the genes identified in this screen were involved in the biofilm lifecycle: flagella synthesis and motility (*flgG*, *H*, *I*, *J*, *K*, *L*, *fliD*, *I*, *K*, *R*, *A*, *motC*, *D*, *Y*, *A*, and *fleN, R, Q*), exopolysaccharide synthesis (*pslE*, *I*, *K*, *L*, and *algC*), cyclic diguanylate (c-di-GMP) synthesis (*siaC* and *D*), quorum sensing (*lasR*), and *cis*-2-decenoic acid synthesis (*dspI*). In addition, multiple transposon insertions were identified in genes related to metabolic pathways, including the leucine/isovalerate catabolic pathway (*liuB* and *C*) and purine metabolism pathway (*purC*, *D*, *E*, *L*, and *M*).

### Interplay between biofilm formation and MV formation in static biofilms

To elucidate the interplay between biofilm and MV biogenesis, biofilm formation of the low MV-producing mutants on microtiter plates was quantified using a CV assay. This assay allowed us to identify the factors associated with the initial attachment in biofilm formation. Mutants related to flagella motility (*flg* genes, *fli* genes, *fle* genes, and *mot* genes) and polysaccharide synthesis (*psl* genes) showed significantly decreased biofilm formation, as well as MV production (Table 3), suggesting that those biofilm-related factors are also involved in increased MV formation. However, *algC*::T8, which are involved in exopolysaccharide synthesis, showed high biofilm-forming ability for unknown reasons. Mutants of genes related to purine metabolism (*purM*, *C*, *L*, *D*, and *E*) commonly showed decreased MV formation, but no common feature in growth or biofilm formation was observed (Table 3), suggesting that decreased MV formation in static culture is not due to differences in biofilm formation capacity in purine metabolism-related mutants. There were also several strains that seemed to impair growth and lack the energy to produce MVs.

### MVs are formed during flagella and Psl-driven attachment

**FIGURE 5:**
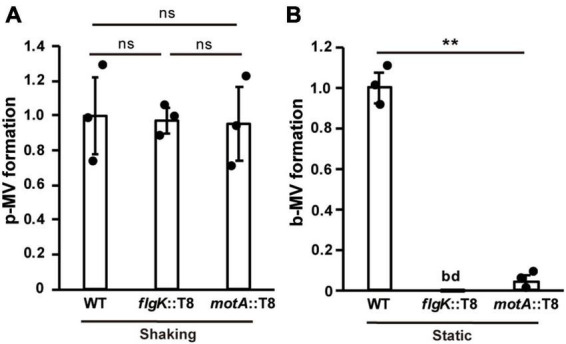
Flagella motility enhances MV formation in biofilms. Planktonic MV (p-MV) and biofilm MV (b-MV) formations of PAO1 wild type (WT), *flgK*::T8, and *motA*::T8 were examined under shaking **(A)** and static biofilm **(B)** conditions, respectively. The amount of vesicles extracted from the supernatants was normalized to the cell density, and each value shown is relative to that of WT. The data are presented as the mean ± SD from three replicates. **P < 0.01; ns, not significant; bd, below the limit of detection. One-way ANOVA followed by Turkey’s multiple comparison tests was used.

To gain further insight into the effects of biofilm-related factors on MV production, we extracted MVs and quantified MV production per bacterial cell in WT and several mutants under shaking (planktonic) and static (biofilm) conditions. The flagellar synthesis-defective mutant (*flgK*::T8) and the motility-defective mutant (*motA*::T8) showed similar MV formation compared to that in the WT under planktonic conditions (Figure 5A), but were significantly repressed under biofilm conditions (Figure 5B), indicating that the induction of MV formation by flagellar motility is a biofilm-specific process.

**FIGURE 6:**
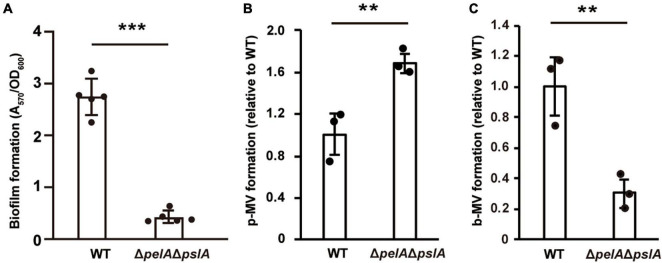
Exopolysaccharides enhance MV formation in static culture. **(A)** Biofilm formation of WT and Δ*pelA*Δ*pslA* on microtiter plates. The amount of biofilm was normalized to the cell density. The data are presented as the mean ± SD from five replicates. ***P < 0.001. Planktonic MV (p-MV) and biofilm MV (b-MV) formations of WT and Δ*pelA*Δ*pslA* were examined under shaking **(B)** and biofilm **(C)** conditions, respectively. The amount of vesicles extracted from the supernatants was normalized to cell densities, and each value shown is relative to that of WT. The data are presented as the mean ± SD from three replicates. **P < 0.01 (*t*-tests).

Next, we examined whether the effect of exopolysaccharides on MV formation is altered by culture conditions. *P. aeruginosa* produces Pel and Psl exopolysaccharides (Ryder et al., 2007). Pel is a glucose-rich, cellulose-like polymer associated with pellicle formation at air-liquid interfaces. Psl is rich in mannose and galactose and is involved in initial attachment (Ghafoor et al., 2011). Biofilm formation in the PAO1 strain is mainly regulated by Psl polysaccharide (Ghafoor et al., 2011; Colvin et al., 2012), but Pel polysaccharide has some influence in the absence of Psl (Tashiro et al., 2014). Therefore, we compared MV formation using a mutant strain Δ*pelA*Δ*pslA*, in which both genes were deleted in a frame. Biofilm formation by this mutant was significantly repressed (Figure 6A). While MV formation in Δ*pelA*Δ*pslA* was slightly increased compared to that in WT under shaking conditions (Figure 6B), it was significantly decreased under static conditions (Figure 6C), indicating that exopolysaccharides play a critical role in biofilm-specific MV formation. When we compared the effect of Pel and Psl using the single mutants, both biofilm formation and b-MV formation were decreased in Δ*pslA* to the same level as the double mutant Δ*pelA*Δ*pslA*, but not in Δ*pelA* (Supplementary Figures 3A, B), suggesting that increased MV formation in PAO1 biofilm is dependent on Psl. Complementation analysis showed that MV formation of Δ*pslA* expressing plasmid-based *pslA* was at the same level as the control (Supplementary Figure 3C).

**FIGURE 7:**
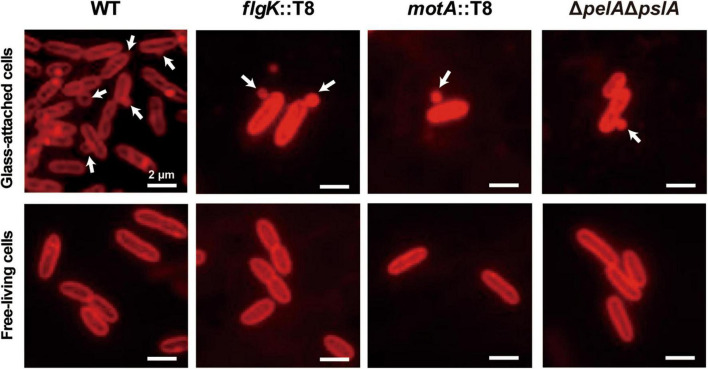
Surface sensing facilitates MV formation. Bacterial cells (PAO1 WT, *flgK*::T8, *motA*::T8, and Δ*pelA*Δ*pslA*) were grown in a static biofilm condition for 2.5 h, and half of coverslips were immersed in each culture during incubation. Glass-attached cells on coverslips **(upper row)** and free-living cells in cultures **(lower row)** were stained with FM4-64. Released vesicles are shown by white arrows. Bar, 2 μm.

To further investigate the effect of flagella motility and exopolysaccharides on MV production, we focused on the early stage of biofilm formation. WT and each mutant were grown for 2.5 h under static conditions, in which coverslips were vertically stood, and glass-attached cells were observed by fluorescence microscopy. Bacterial attachments were repressed in low-biofilm forming mutants (*flgK*::T8, *motA*::T8, and Δ*pelA*Δ*pslA*), and staining Psl-specific HHA-lectin revealed that Psl was actively formed in WT, but not synthesized in *flgK*::T8, as well as Δ*pelA*Δ*pslA* (Supplementary Figure 4). The Psl was detected in the glass-attached cells of *motA*::T8, but the signal level was significantly lower than that of WT. When bacterial cells attached to glass were stained with FM4-64, active MV production was observed on the coverslips in both WT and mutants (Figure 7). MVs on the cellular surface were rarely observed in free-living cells, not attached to the glass, in any of the samples (Figure 7). These results suggest that flagellar motility and Psl indirectly enhance MV formation as a result of increasing contact between bacterial cells and surfaces, and surface attachment activates MV blebbing.

## Discussion

Membrane vesicles are a component of the biofilm matrix, and their close relationship has long been the focus of attention (Schooling and Beveridge, 2006; Wang et al., 2015). Previous reports showed that the production of MVs is significantly increased under biofilm conditions in several bacteria (Schooling and Beveridge, 2006; Yonezawa et al., 2009; Grande et al., 2017), and MVs regulate biofilm morphology (Esoda and Kuehn, 2019; Cooke et al., 2020); however, the mechanisms of increased MVs in biofilms are not fully understood. In this study, we comprehensively identified the factors involved in enhanced MV production in static biofilms in LB medium by screening a transposon mutant library from the *P. aeruginosa* PAO1 strain. We identified 55 genes required for enhanced MV formation in static culture where biofilm is formed.

The liquid static culture used in this study showed a significant enhancement in MV formation compared to the shaking culture conditions (Figure 1). Furthermore, this static condition was suitable for screening altered MV-forming mutants and the relationship between initial attachment and MV formation, in contrast to the colony-biofilm. Observation of the static culture showed thick biofilm formation with MVs at the pellicle and edge of the cover glass (Figure 3). Based on a previous report, it is estimated that the fibrous substance entangling cells and MVs is Psl (Byrd et al., 2010), the main component of the biofilm matrix in *P. aeruginosa*. Psl appears to be used by cells to aggregate and adhere to surfaces.

Notably, the observation of the bacterial envelope in the biofilm state by QFDE-EM revealed several curvatures of OM and the separation of OM and IM (Figures 4B, C and Supplementary Figures 2E, F). Such bacterial plasmolysis, which causes a large gap in the periplasmic space, has been observed in a hypervesiculating *E. coli* mutant (Δ*mlaE*Δ*nlpI*) (Ojima et al., 2021) and under hypervesiculating conditions (presence of 1% glycine) (Aktar et al., 2021), suggesting that plasmolysis is one of the key factors for increased MV formation in biofilms. Furthermore, the MV protein composition is significantly different between p-MVs and b-MVs (Figure 2D and Table 2), and biofilm-specific regulation drives enhanced MV formation.

Using a transposon mutant library of PAO1, we isolated 66 low-MV producing mutants in *P. aeruginosa* biofilms, but no high-MV producing mutant strain was obtained, suggesting that the static biofilm is high-vesiculating condition in *P. aeruginosa* (Table 3). This result is different from a previous study that screened altered MV-forming *E. coli* mutants; only a few low-vesiculation mutants were obtained, although there were many hypervesiculating mutants which lack components of the OM, peptidoglycan synthesis, or the σ*^E^* envelope stress pathways (McBroom et al., 2006). In the present study, we found that mutations in flagellar motility, exopolysaccharide synthesis, metabolic pathways, two-component systems, and signal synthesis reduced MV production in static biofilms. Examining the effect of these genes constructing mutations with non-polar effect will yield further insight regarding the contribution of specific factors to MV formation under biofilm conditions.

As both flagellar motility and exopolysaccharide (Psl) synthesis are involved in the initial attachment of biofilm formation (Byrd et al., 2009; Leid et al., 2009), we hypothesized that surface attachment triggers MV formation under biofilm conditions. To test this hypothesis, fluorescence microscopy observations of the early stage of WT and each mutant biofilm cells with membrane staining was conducted. It revealed that cells attached to coverslips produced MVs more actively than free-living cells in liquid static culture (Figure 7). Flagella motility and exopolysaccharide synthesis-defective mutants, which showed low MV production in static culture, also produced MVs on coverslips, suggesting that surface sensing is closely related to MV production. This observation is consistent with a previous result by Byrd et al. (2010); protrusions approximately 100 nm in diameter were observed on the membrane of adherent *P. aeruginosa* PAO1, but a similar structure was not found in its Δ*psl* mutant. During initial attachment, bacterial cells sense surface attachment through various pathways, including pili, signal receptions, two-component regulatory systems, and envelope stress on the cell wall (O’Toole and Wong, 2016; Chang, 2017). Although the details of the mechanism are unclear, it appears that these factors are intimately involved in the induction of OM curvature and blebbing.

We also found that several mutants related to biofilm formation and maintenance showed low vesiculation in static culture. Two mutants with disruptions in *siaC* and *siaD*, which are genes important for c-di-GMP synthesis (Chen et al., 2020), were identified with low vesiculation and low biofilm formation. c-di-GMP is an intracellular signaling molecule and a key signal for *P. aeruginosa* to switch from planktonic to attachment states during the early stage of biofilm formation, and extracellular polysaccharide synthesis is enhanced with increased c-di-GMP levels (Romling et al., 2013). While the direct relationship has not been fully investigated, the intracellular level of c-di-GMP may be associated with MV production in biofilms.

In contrast, mutations in *lasR* and *dspI* resulted in low MV formation, although the ability to form biofilms were not significantly different from WT. Las is one of the quorum sensing systems using *N*-(3-oxo-dodecanoyl) L-homoserine lactone (3-oxo-C12-HSL), and the LasR and 3-oxo-C12-HSL complex activates transcription related to virulence factors (Parsek and Greenberg, 2000). DspI is a putative enoyl-coenzyme A (CoA) hydratase/isomerase that is involved in the synthesis of *cis*-2-decenoic acid (CDA), which regulates biofilm dispersion and the transcription of many genes related to motility and virulence (Amari et al., 2013; Rahmani-Badi et al., 2015). Since these mutants showed low production of MVs without suppressing biofilm formation, it is possible that the genes are involved in the induction of MV formation in biofilms and should be analyzed in the future.

There were six low-vesiculation mutants of purine nucleotide metabolism and their growth was impaired. The de novo purine is essential for both DNA and RNA production, with purines playing an important role in energy generation (ATP and GTP) and signal molecule synthesis (c-di-GMP, cAMP, and (p)ppGpp) (Jewett et al., 2009). Energy storage and signaling or global changes in metabolism are likely to be involved in MV production in biofilms.

Furthermore, three low-vesiculation mutants of the leucine/isovalerate utilization (Liu) pathway had no effect on growth. Acetyl-CoA, the end product of the Liu pathway, is used for the tricarboxylic acid cycle and glyoxylate cycle (Hoschle et al., 2005; Diaz-Perez et al., 2018). Leucine is one of the biofilm-promoting amino acids in *P. aeruginosa* (Bernier et al., 2011), but the effect of these genes on biofilm formation was not significant in this study. These factors are also potential candidates for direct involvement in MV formation in biofilms.

Biofilm formation is the optimal growth mode for *P. aeruginosa* when chronically infecting host epithelial cells, and enhanced MV formation in this state is a sophisticated infection strategy to efficiently deliver virulence factors to the host. Indeed, FptA, EprS, OprD, and OprQ were abundant in b-MVs, while PaAP was a major component of p-MVs. OprD, which is an important porin for the entry of carbapenem and imipenem antibiotics into cells, is abundant in PAO1 biofilm cells and clinical *P. aeruginosa* isolates (Park et al., 2014a), and is also rich in MVs derived from colony biofilms (Toyofuku et al., 2012; Couto et al., 2015; Park et al., 2015). OprD functions as a receptor for laminin, a major component of the lung extracellular matrix, and in *P. aeruginosa* it strongly adheres to basal lamina (Paulsson et al., 2019), suggesting that MVs contribute to stabilizing biofilm formation in lung cells during infection. EprS is an autotransporter (AT) protein with serine protease activity that is secreted by the type V secretion system (T5SS) and induces inflammatory responses (Kida et al., 2013). Thus, b-MVs possess a high abundance of proteins that contribute to cell adhesion and inflammation, respectively, and their pathogenicity is crucial to chronic infection.

Although the underlying mechanisms of enhanced MV production in bacterial biofilms are not fully understood, this study revealed significant findings using *P. aeruginosa* static biofilms. Plasmolysis and OM curvature occur in biofilm cells, and it seems that blebbing is one of the key factors to release MVs actively from OM in static biofilms. In addition, we identified factors involved in enhanced MV production in biofilms. Flagella motility and Psl synthesis indirectly control MV formation in biofilms, and the attachment during the early stage of biofilm formation may be the switch for MV blebbing. Future work will seek to elucidate the mechanisms and factors that directly influence MV production in biofilms.

## Data availability statement

The datasets presented in this study can be found in online repositories. The names of the repository/repositories and accession number(s) can be found in the article/Supplementary material.

## Author contributions

MK and YT contributed to the conception and prepared the manuscript. MK, TS, SU, MS, KK, and YT designed the experiments. MK, TS, and SU performed the analyses of vesicle. MK, SU, MT, KH, YOT, MM, and RN contributed the microscopic observations. TS, KH, and MM contributed the protein analyses. MK performed the construction and analyses of transposon mutants. MS, KK, HF, and YT supervised the work. All authors reviewed the manuscript and approved the submitted version.
