# Supplementary material for: Identification of genes involved in enhanced membrane vesicle formation in Pseudomonas aeruginosa biofilms: surface sensing facilitates vesiculation
Source: Front Microbiol. 2023 Dec 1;14:1252155. doi: 10.3389/fmicb.2023.1252155 (PMC10722149; doi:10.3389/fmicb.2023.1252155)
Supplement: Supplementary file 1 [file Data_Sheet_1.pdf]

Supplementary Material

Identification of genes involved in enhanced membrane vesicle formation in *Pseudomonas aeruginosa* biofilms: surface sensing facilitates vesiculation.

Mizuki Kanno[1,2], Takuya Shiota[2], So Ueno[2], Minato Takahara[2], Keisuke Haneda[3], Yuhei O Tahara[4], Masaki Shintani[1,2,3,5,6], Ryoma Nakao[7], Makoto Miyata[4], Kazuhide Kimbara[1,2,3], Hiroyuki Futamata[1,2,3,5], Yosuke Tashiro[1,2,3,8]*

[1] Graduate School of Science and Technology, Shizuoka University, Hamamatsu, 432-8561, Japan

[2] Department of Engineering, Graduate School of Integrated Science and Technology, Shizuoka University, Hamamatsu, 432-8561, Japan

[3] Department of Applied Chemistry and Biochemical Engineering, Faculty of Engineering, Shizuoka University, Hamamatsu, 432-8561, Japan

[4] Graduate School of Science, Osaka Metropolitan University, Osaka, 558-8585, Japan.

[5] Research Institute of Green Science and Technology, Shizuoka University, Shizuoka, 422-8529, Japan

[6] Japan Collection of Microorganisms, RIKEN BioResource Research Center, Tsukuba, 305-0074, Japan.

[7] Department of Bacteriology, National Institute of Infectious Diseases, Shinjuku-ku, Tokyo, Japan.

[8] JST PRESTO, Kawaguchi, 332-0012, Japan

*** Correspondence:**
Yosuke Tashiro
tashiro.yosuke@shizuoka.ac.jp

Table S1. Primers used in this study.

Primers	Sequence (5'-3')	References
PA0628F	GAGCATGTCGACCGGCACCTCTATACCAGC	This study
PA0630R	GCCTCCTCCAGTTGCGGACGATAATGGCGG	This study
CEKG2A	GGCCACGCGTCGACTAGTACNNNNNNNNNNAGAG	(Jacobs et al., 2003)
CEKG2B	GGCCACGCGTCGACTAGTACNNNNNNNNNNACGCC	(Jacobs et al., 2003)
CEKG2C	GGCCACGCGTCGACTAGTACNNNNNNNNNNGATAT	(Jacobs et al., 2003)
CEKG4	GGCCACGCGTCGACTAGTAC	(Jacobs et al., 2003)
lacZ-211	TGCGGGCCTCTTCGCTATTA	(Jacobs et al., 2003)
lacZ-148	GGGTAACGCCAGGGTTTTCC	(Jacobs et al., 2003)
pslA-F-Hind3	CGGTATCGATAAGCTGGACTGCCCGTGATCGGCAGAGCAAAC	This study
pslA-R-Xba1	TGGCGGCCGCTCTAGGCGACGGCGTTCATCAGTAGACTTCCT TGGTC	This study

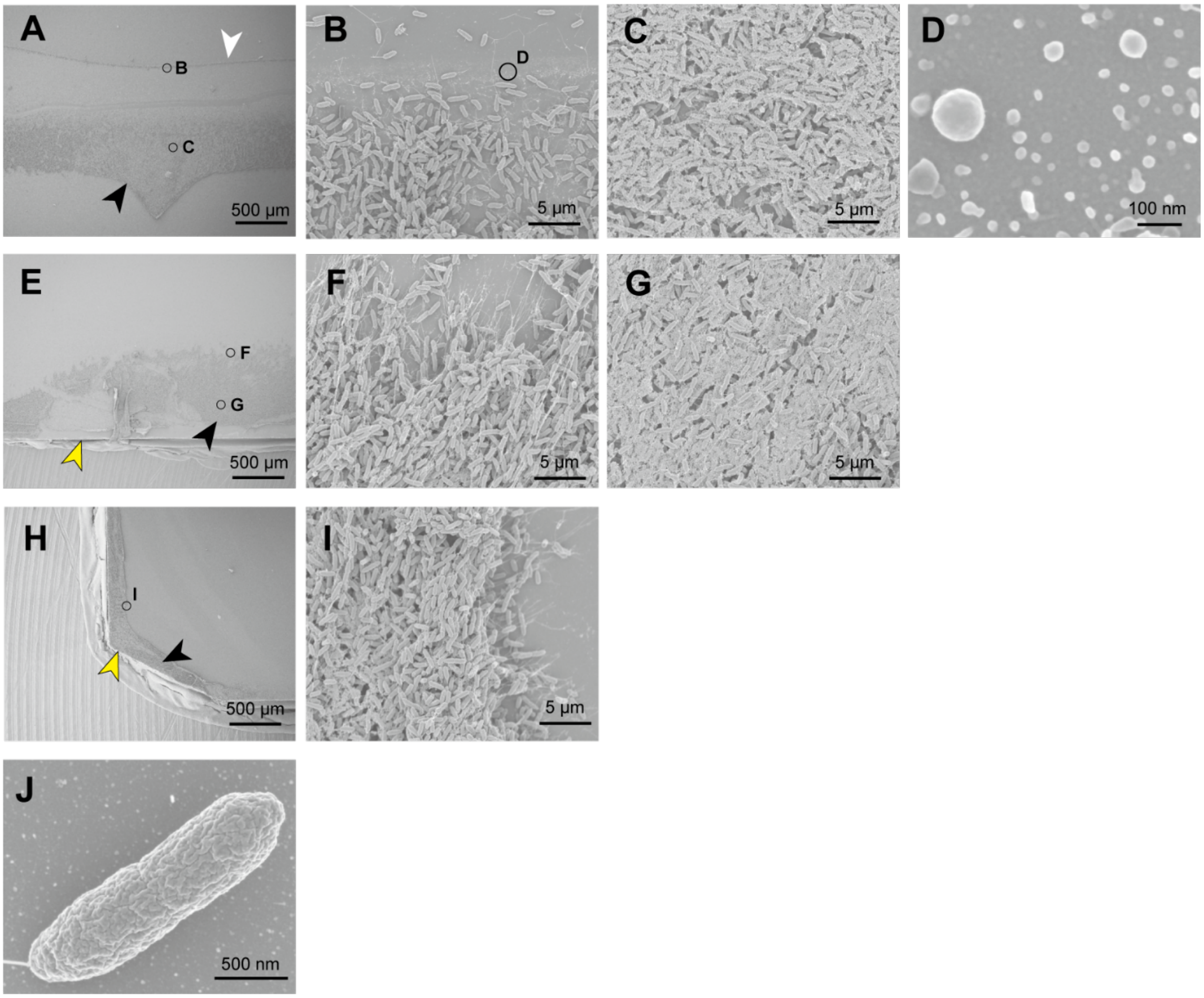

Supplementary Figure 1. Bacterial cells inside biofilm visualized by SEM. The illustration of visualized points in the static culture is shown in Figure 2A. (A–D) The air-liquid interface of the vertically stood glass. A, a field image; B, an image of the upper layer; C, dense bacterial cells in biofilm; D, vesicles attached to the glass. Observation areas of B, C, and D are shown in circles. White and black arrows indicate thin and thick biofilms, respectively. (E–G) The bottom of the vertically standing glass. E, a field image; F, an edge of thick biofilm; G, dense bacterial cells in the thick biofilm. Observation areas of F and G are shown in circles. (H, I) The bottom of the horizontally placed glass. H, a field image; I, an edge of thick biofilm. The black arrow indicates thick biofilms and the yellow indicates the glass edge. (J) The planktonic cell under a shaking culture.

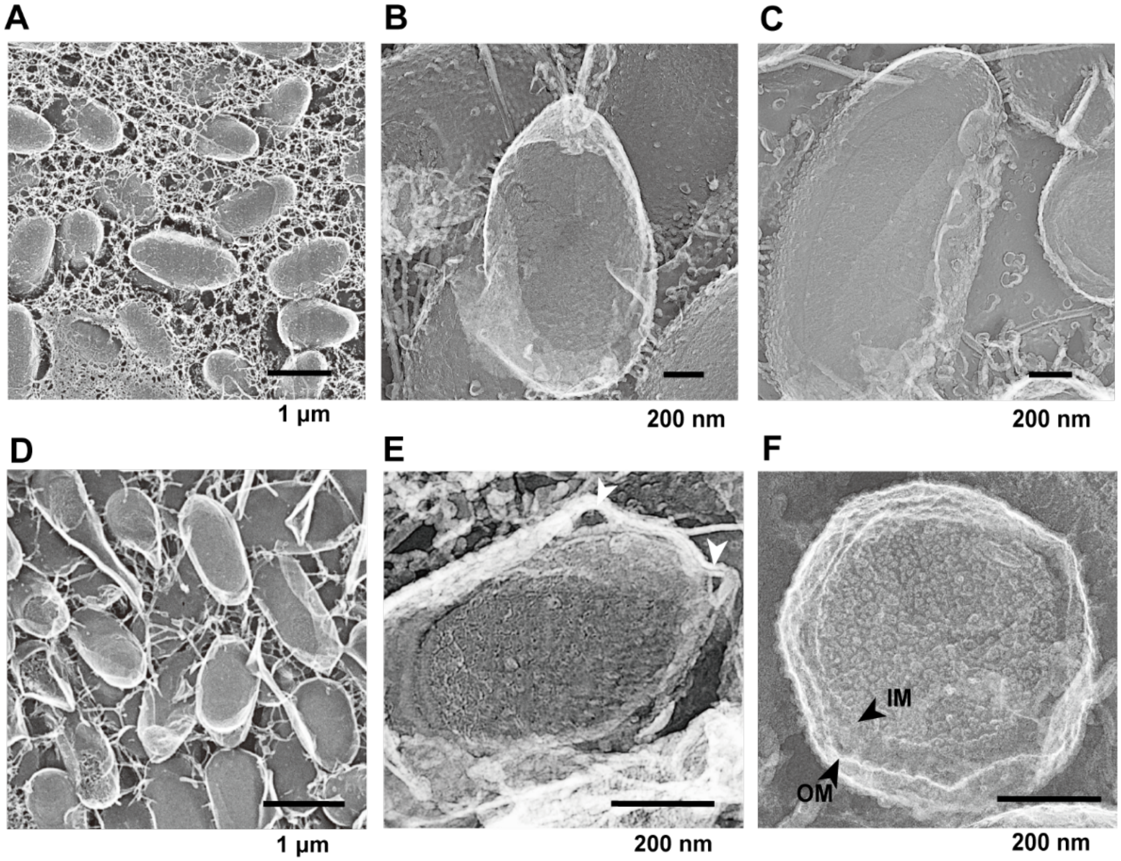

Supplementary Figure 2. QFDE micrographs of *P. aeruginosa* PAO1 cells. Field images of cells grown under the shaking condition (A-C) and the static condition (D-F) are shown. (E, F) Images of cells grown under the static condition for 12 h. The protrusion of the OM is shown by white arrows (E). A broad space between the OM and IM (black arrows) was observed in some cells (F). OM, Outer membrane; IM, inner membrane.

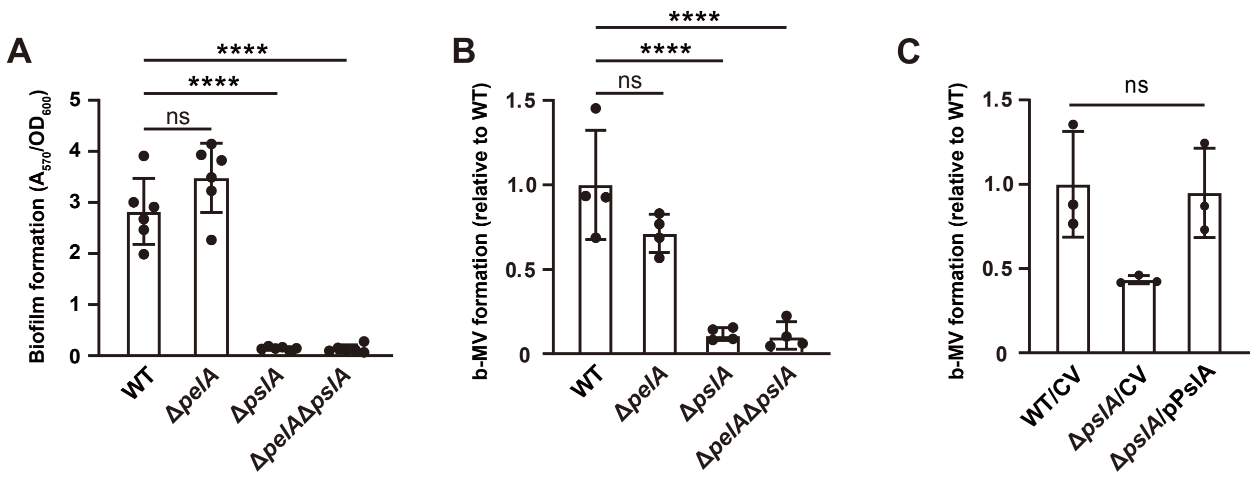

Supplementary Figure 3. Psl enhances MV formation under the static biofilm conditions. (A) Biofilm formation of WT, Δ*pelA*, Δ*pslA* and Δ*pelA*Δ*pslA* on microtiter plates. The amount of biofilm was normalized to the cell density. The data are presented as the mean standard deviation from six replicates. (B) Biofilm MV (b-MV) formations of WT, Δ*pelA*, Δ*pslA,* and Δ*pelA*Δ*pslA* were examined under static conditions. (C) Biofilm MV (b-MV) formations of WT-CV, Δ*pslA*-CV, and Δ*pslA*-pPslA were examined under static conditions. The amount of vesicles extracted from the supernatants was normalized to cell densities, and each value shown is relative to that of the control. CV shows a control vector pBBR1MCS-5. The data are presented as the mean ± standard deviation from three or four replicates. ****, $P < 0.0001$; ns, not significant (one-way ANOVA with Turkey's multiple comparisons for more than three groups).

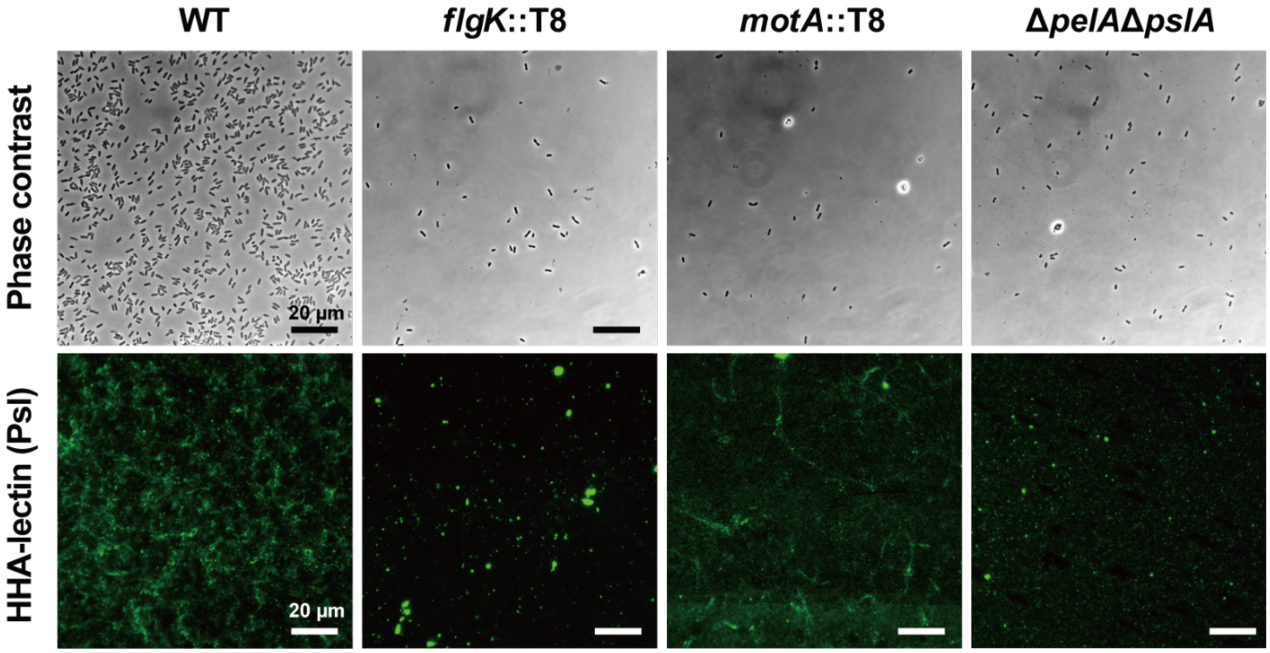

Supplementary Figure 4. Bacterial attachment to the glass. Bacterial cells (WT, *flgK*::T8, *motA*::T8 and Δ*pelA*Δ*pslA*) were grown under static biofilm conditions for 2.5 h with immersing half of coverslips. Exopolysaccharide Psl was stained with HHA-FITC lectin. Attached bacterial cells and secreted Psl on coverslips were observed by phase contrast (upper row) and fluorescent microscopy (lower row). Bar = 20 μm.

REFERENCES

Jacobs, M.A., Alwood, A., Thaipisuttikul, I., Spencer, D., Haugen, E., Ernst, S., Will, O., Kaul, R., Raymond, C., Levy, R., Chun-Rong, L., Guenthner, D., Bovee, D., Olson, M.V., and Manoil, C. (2003). Comprehensive transposon mutant library of *Pseudomonas aeruginosa*. *Proc Natl Acad Sci USA* 100**,** 14339-14344.
